# Supplementary material for: Intracellular hydrogelation preserves fluid and functional cell membrane interfaces for biological interactions
Source: Nat Commun. 2019 Mar 5;10:1057. doi: 10.1038/s41467-019-09049-5 (PMC6401164; doi:10.1038/s41467-019-09049-5)
Supplement: Supplementary file 1 — Supplementary Information [file 41467_2019_9049_MOESM1_ESM.pdf]

Supplementary Information:

Intracellular hydrogelation preserves fluid and functional cell membrane interfaces for biological interactions

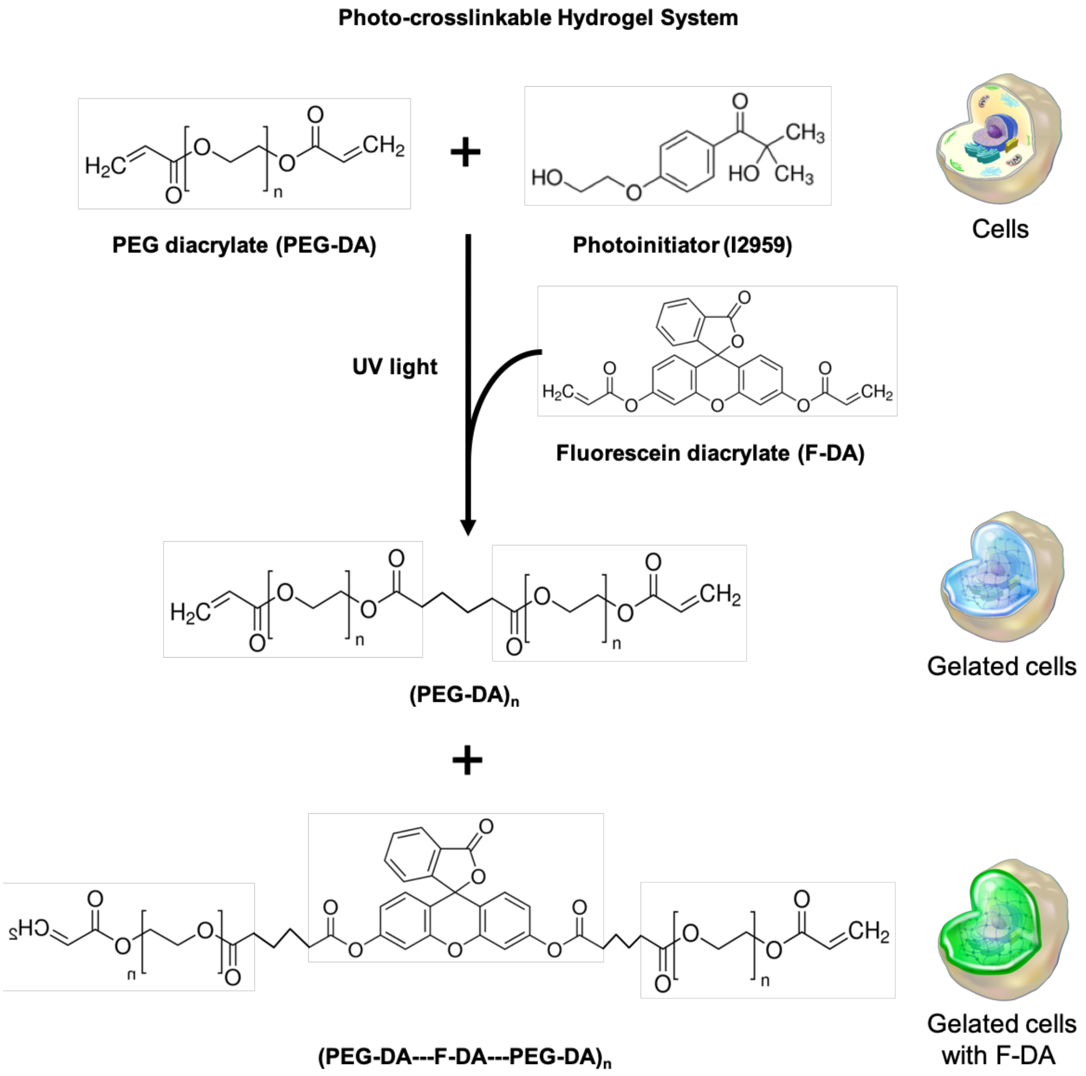

Supplementary Figure 1. Photo-crosslinkable hydrogel system for intracellular hydrogelation. PEG-DA-based hydrogels were formed by photopolymerization of PEG-DA solution with a photoinitiator, I2959. Under ultraviolet (UV) exposure, I2959 absorbs light and activates PEG-DA monomers to form covalently bonded hydrogel networks. The hydrogel can be covalently imbued with green fluorescence by incorporating fluorescein diacrylate into the gelation mixture.

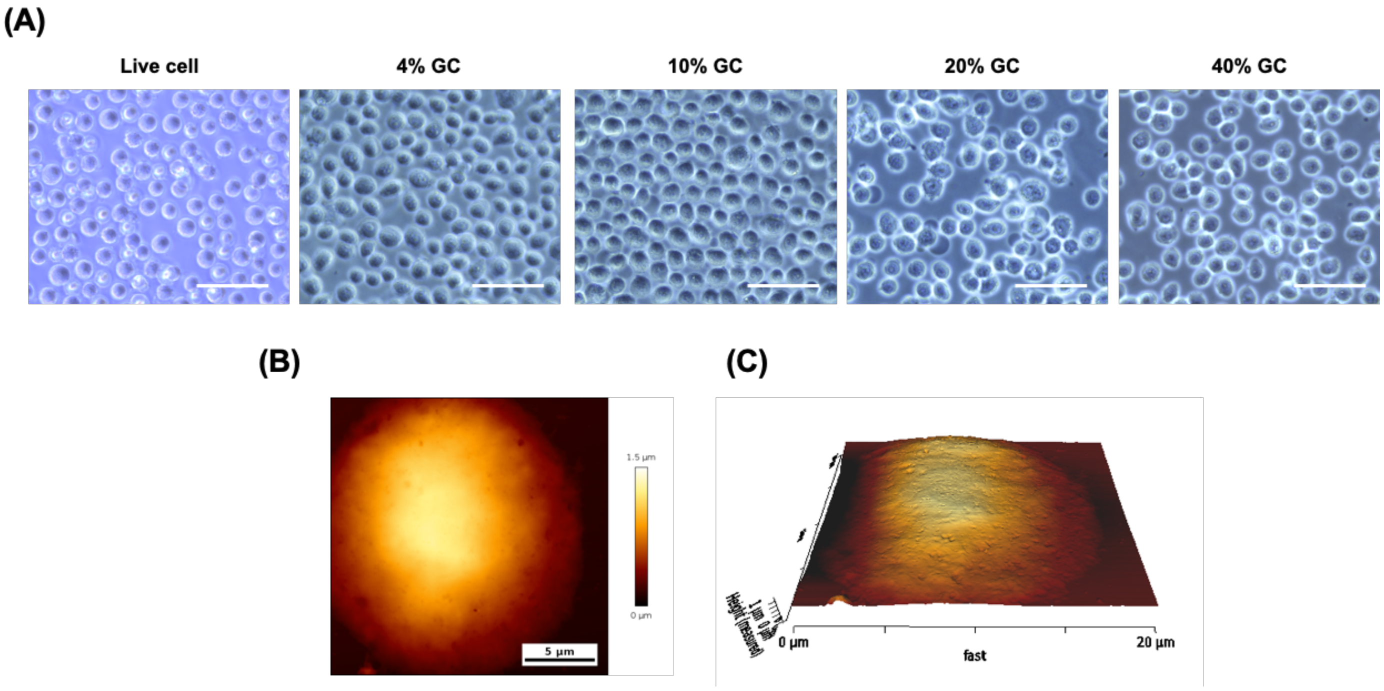

Supplementary Figure 2. Microscopy observation of intracellularly gelated HeLa cells with various PEG-DA densities. (A) HeLa cells gelated in the presence of I2959 and different concentrations of PEG-DA showed similar morphology to live HeLa cells. Scale bars = 40 µm. **(B)(C)** Topographical map of a gelated HeLa cell (20 wt% of PEG-DA) constructed by atomic force microscopy.

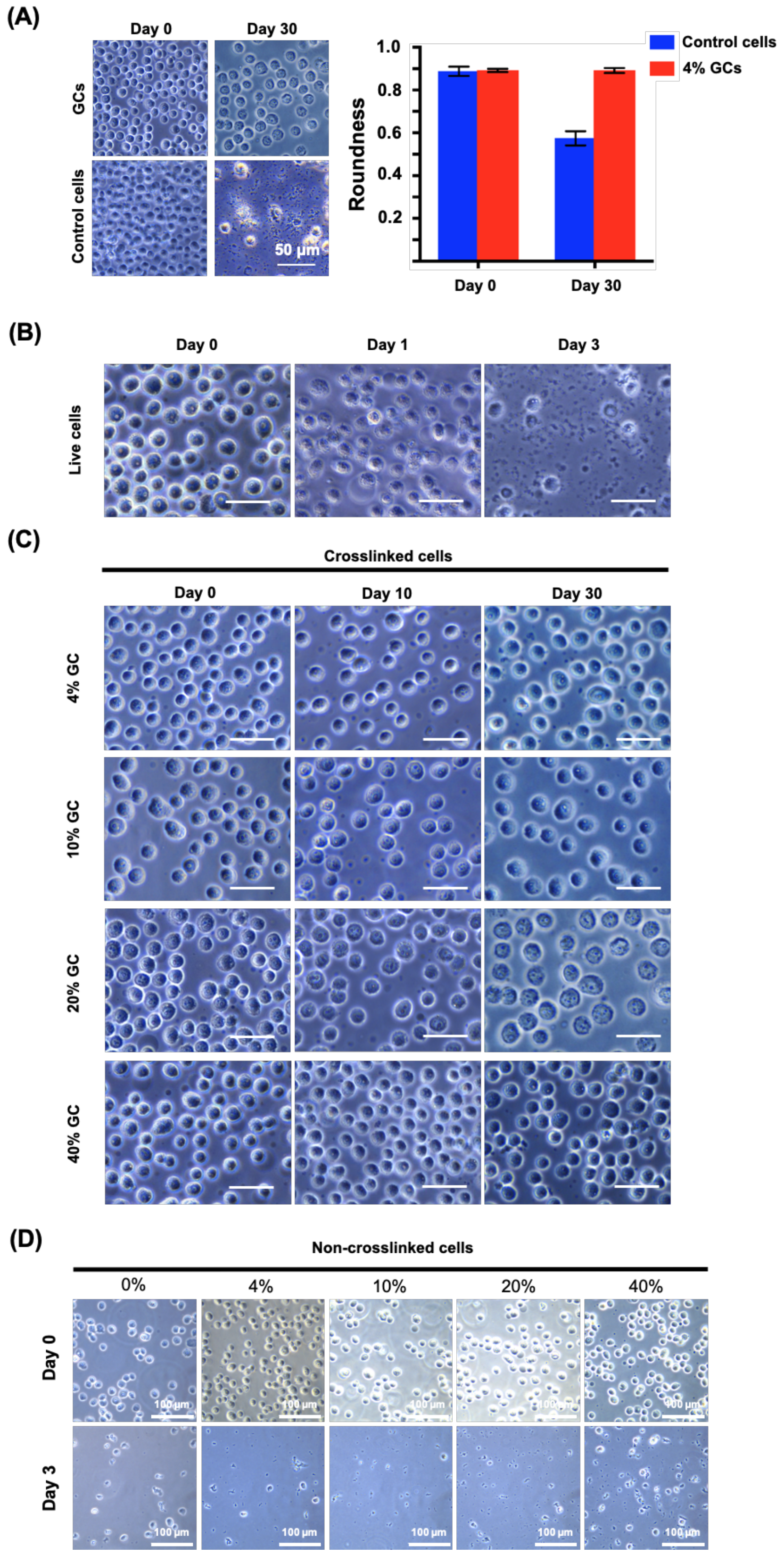

Supplementary Figure 3. Gelated HeLa cells, but not non-crosslinked cells, maintain morphology in PBS. (A) Quantification of cellular roundness of 4 wt% GCs and control cells suspended in PBS for 0 and 30 days. **(B)** Non-gelated HeLa cells disintegrated within 3 days upon suspension in PBS. **(C)** Gelated HeLa cells prepared with 4 to 40 wt% of PEG-DA retained their morphology over a 30-day observation period in PBS. Scale bars = 30 µm. **(D)** HeLa cells infused with hydrogel monomers without UV crosslinking showed morphological alterations within 3 days.

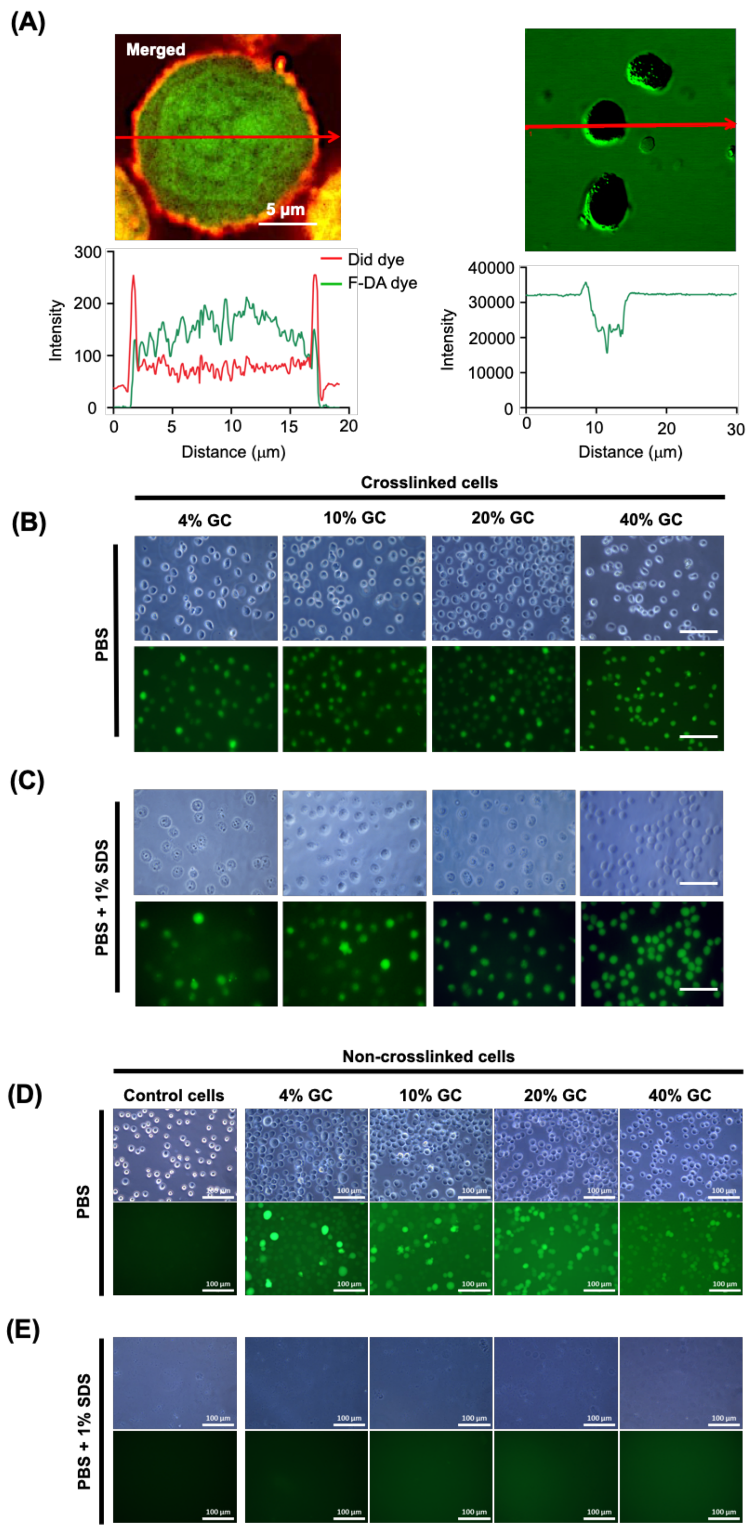

Supplementary Figure 4. Gelated HeLa cells with fluorescein-diacrylate show a fluorescent interior resistant to solubilization by detergent. (A) Fluorescence quantification of DiD-stained 20 wt% GC and 4 wt% GC suspended in fluorescein solution. **(B)** HeLa cells were gelated in the presence of I2959 and different concentrations of PEG-DA with fluorescein-DA. The resulting gelated cells have a fluorescent interior. **(C)** Intracellular gelation was validated upon dissolution of gelated HeLa cells by sodium dodecyl sulfate (SDS). Fluorescent hydrogel matrices remained following the solubilization. Scale bars = 50 µm. **(D)** HeLa cells were infused with I2959 and different concentrations of PEG-DA with fluorescein-DA without UV exposure. **(E)** Without UV crosslinking, the PEG-DA infused cells were completely dissolved by SDS. Scale bars = 100 µm.

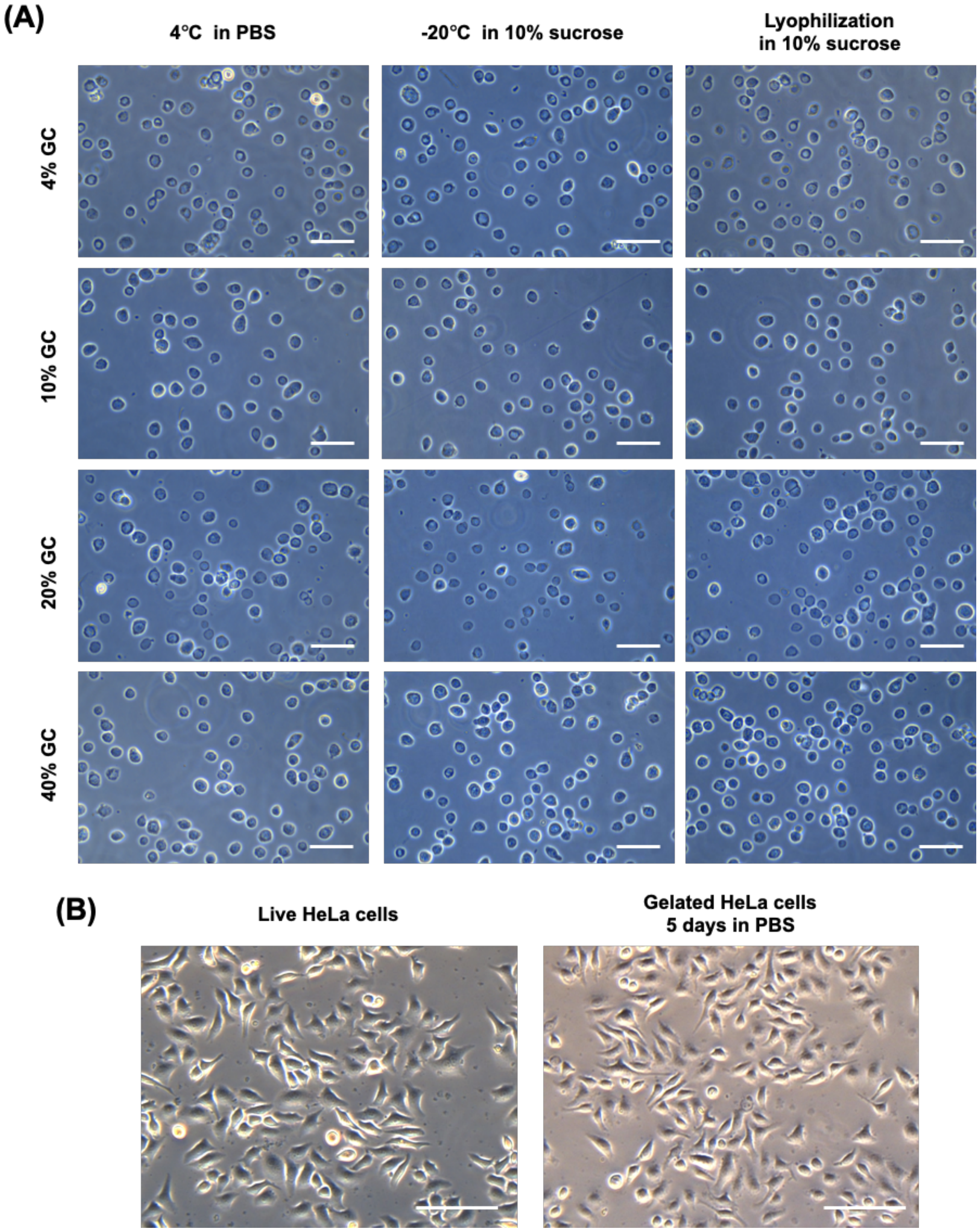

Supplementary Figure 5. Gelated cells under different storage conditions and gelated adherent cells. (A) Gelated HeLa cells prepared with 4 to 40 wt% PEG-DA were stored in PBS at 4°C, frozen in 10% sucrose at -20°C, or lyophilized in 10% sucrose. After 72 hr of storage, the gelated cells (GCs) were observed upon thawing or resuspension in water. No discernable change in morphology was observed with the GCs under the different storage conditions. Scale bars = 50 µm. **(B)** Gelated adherent HeLa cells (20 wt% PEG-DA) in PBS retain their elongated structure after 5 days of observation. Scale bars = 50 µm.

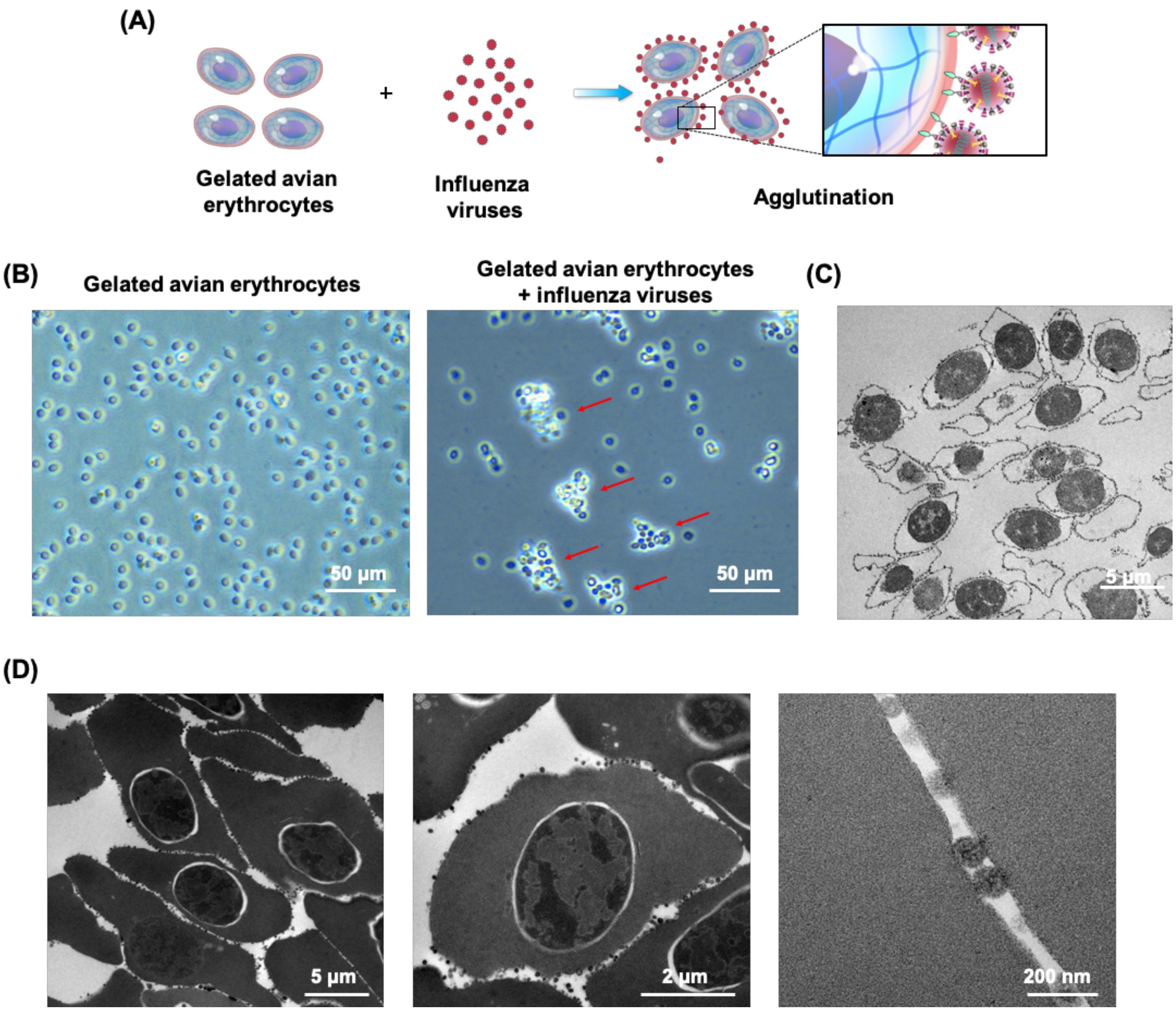

Supplementary Figure 6. Gelated avian erythrocytes readily agglutinate in the presence of influenza viruses. (A) A schematic illustration showing the interaction between influenza viruses and gelated avian erythrocytes. **(B)** Microscopy of gelated avian erythrocytes in the absence (left) and the presence of influenza viruses (right). Red arrows indicate agglutinated cells. **(C)** A TEM cryosection image shows the agglutinated gelated avian erythrocytes in the presence of influenza viruses. **(D)** TEM cryosection images show influenza virus binding on normal avian erythrocytes.

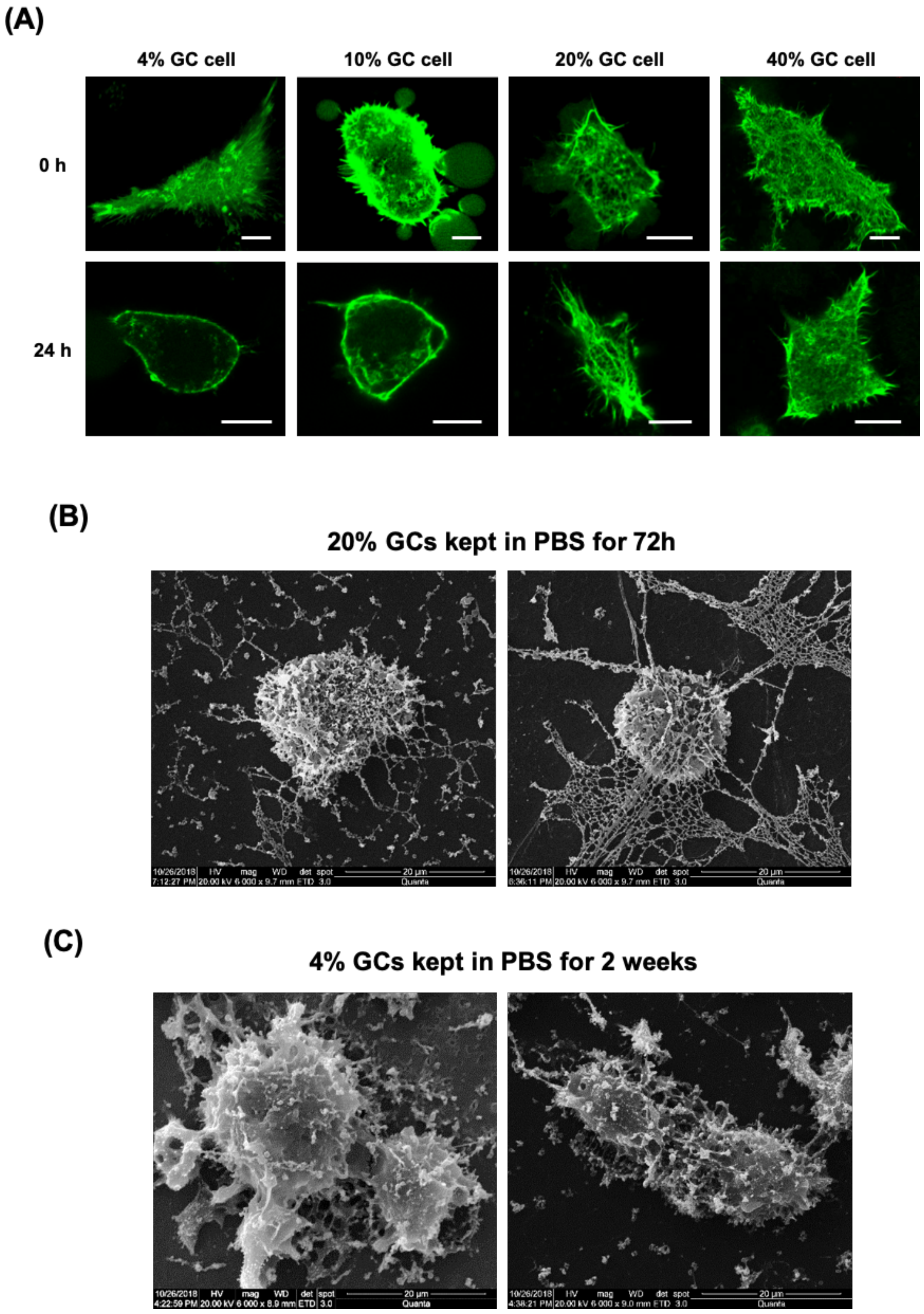

Supplementary Figure 7. GCs preserve cytoskeletal features and membrane ruffles. (A) Fluorescence images of actin-GFP-expressing, gelated HeLa cells (4 to 40 wt%) show preservation of actin filaments following intracellular gelation (upper panel). After 24 h of storage in PBS, actin filaments remained distinguishable in 20 wt% and 40 wt% GCs (lower panels). The images show the gelation process did not disrupt the cytoskeletons. Scale bars = 10 μm. **(B)(C)** Cryogenic scanning electron microscopy (Cryo-SEM) images show that gelated HeLa cells retain ruffled membrane features over extended period.

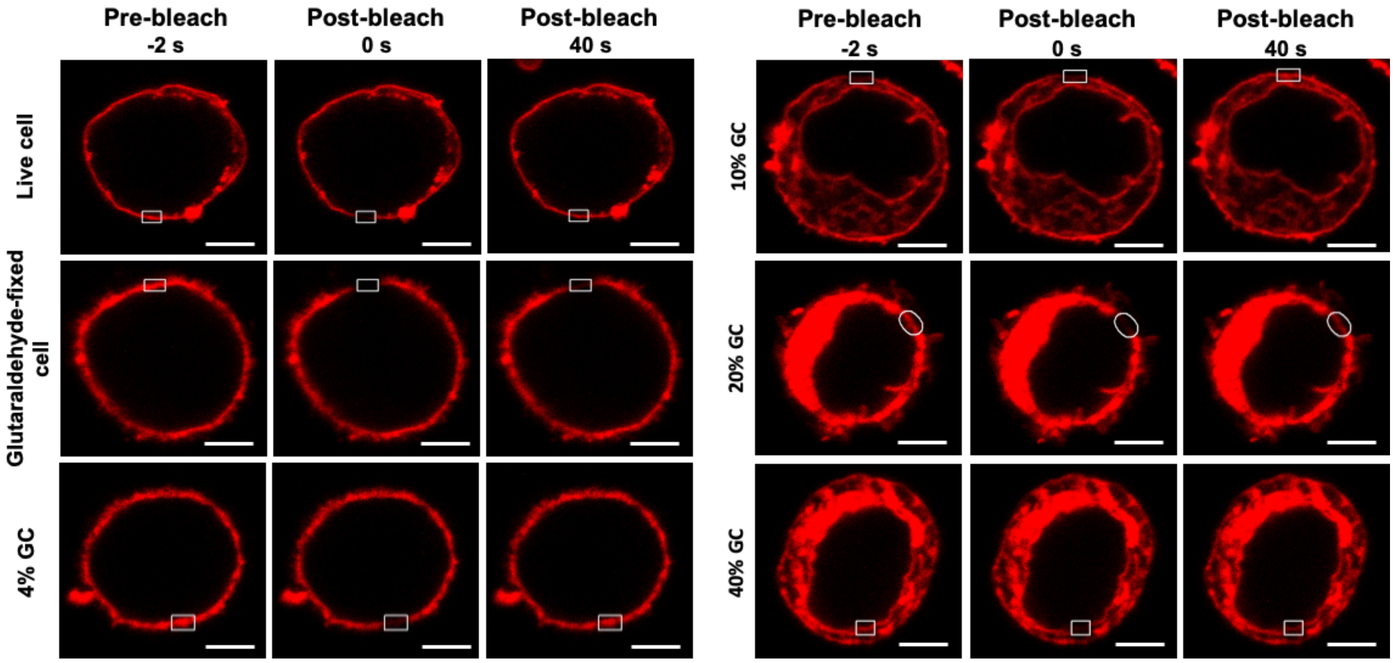

Supplementary Figure 8. Examination of membrane fluidity in gelated cells (GCs). Live HeLa cells, glutaraldehyde-fixed HeLa cells, and gelated HeLa cells of various PEG-DA densities were stained with DiD, and their membrane fluidity was evaluated by FRAP. Representative fluorescence images show the DiD fluorescence before, immediately after, and 40 s after photobleaching. White rectangles indicate the photobleached region of interest. Scale bars = 5 µm.

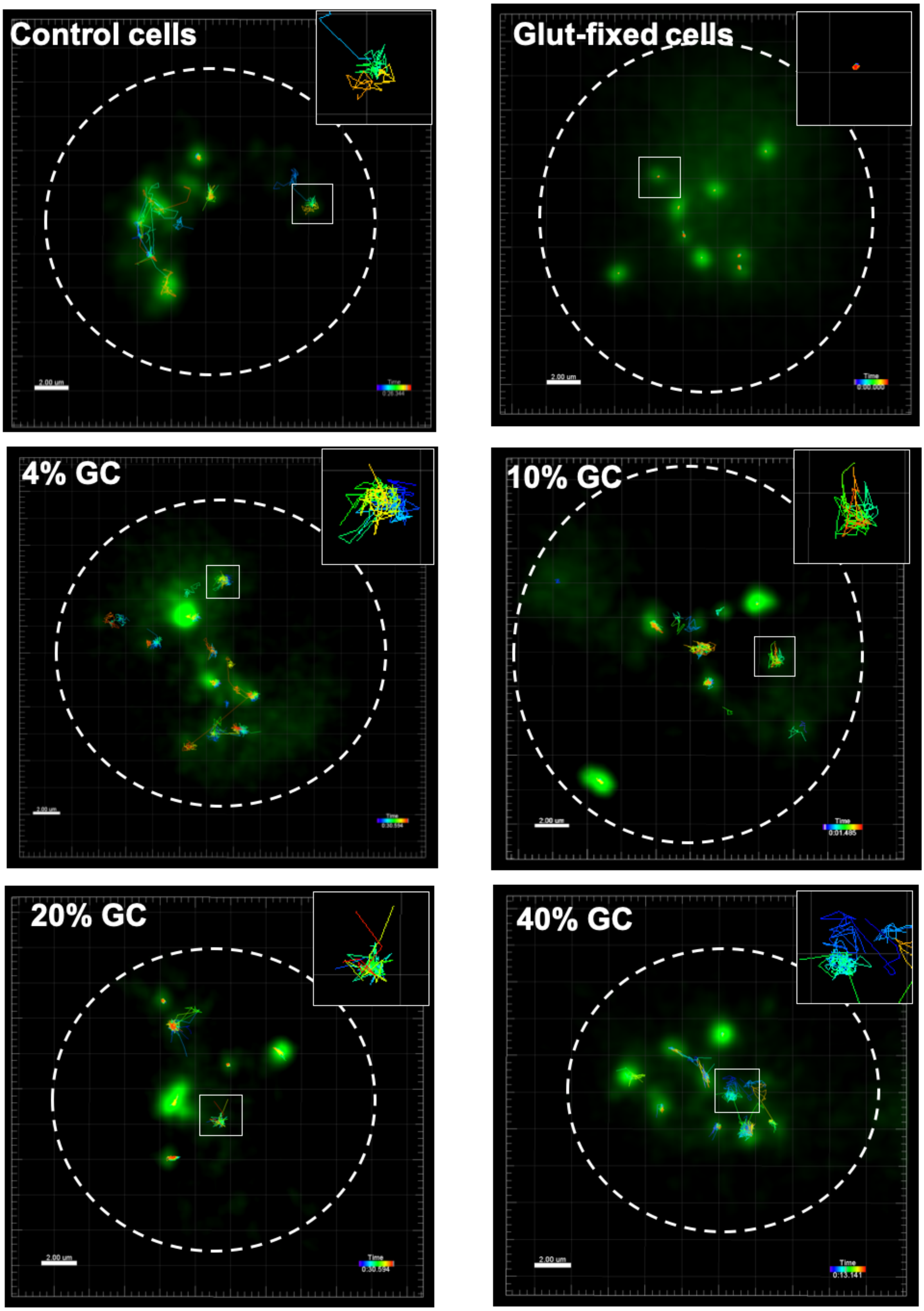

Supplementary Figure 9. TIRF observation and tracking of mobile CD80-GFP in GCs. Lateral mobility of CD80-GFP was observed in transfected HeLa cells using a TIRF microscope. GCs were prepared using 4 to 40 wt% of PEG-DA. Non-gelated HeLa cells having undergone a single freeze-thaw cycle were immediately monitored as a control. A negative control was prepared with glutaraldehyde-fixed cells. Dashed lines indicate the cell boundary and white squares highlight the fluorescence tracking trajectories. Scale bar = 2 μm.

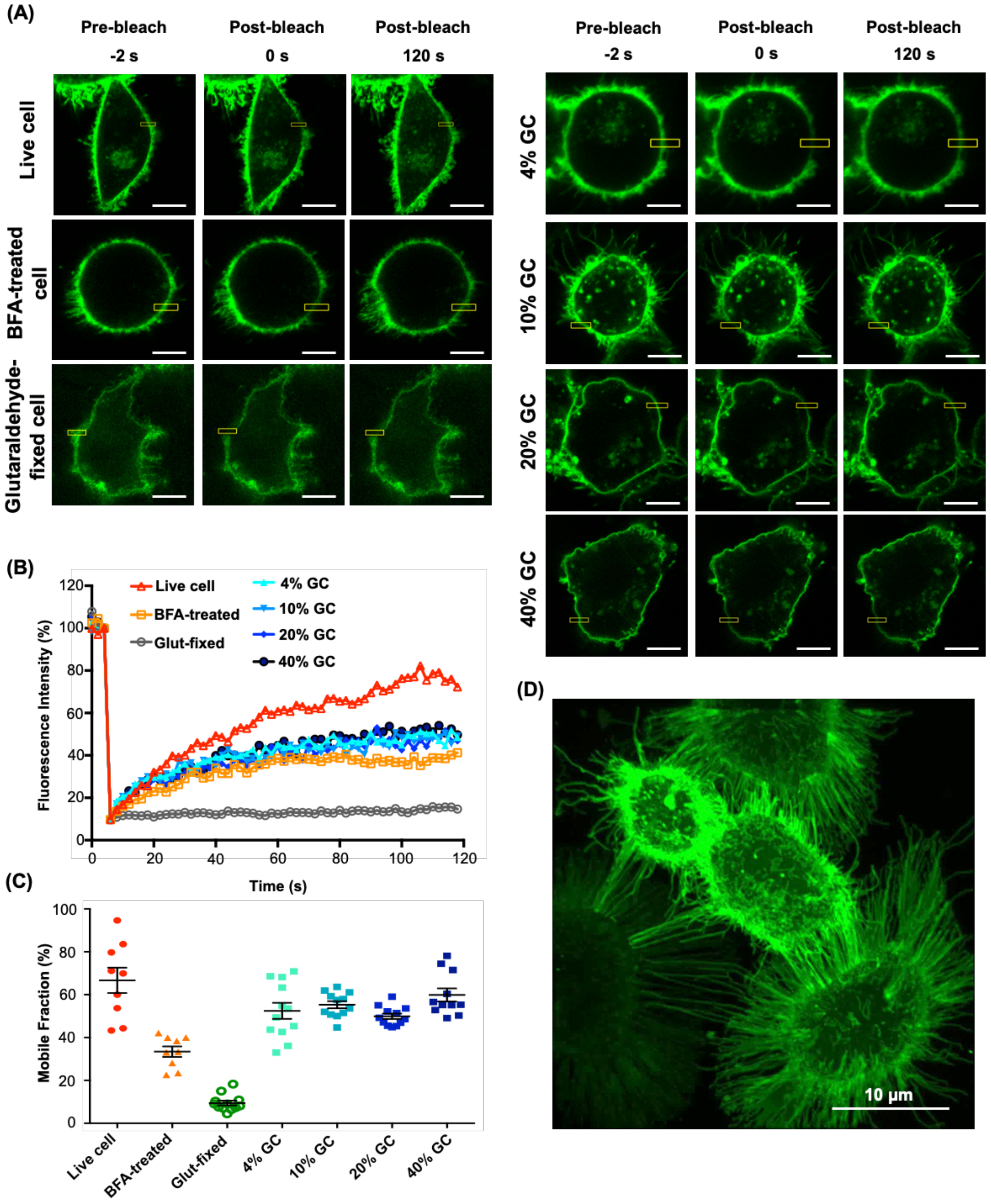

Supplementary Figure 10. CD80 transmembrane proteins remain mobile on GCs. (A) HeLa cells transfected with mouse CD80-GFP plasmid were treated with brefeldin A (BFA, a membrane trafficking inhibitor), fixated with glutaraldehyde, or gelated with different concentrations of PEG-DA prior to FRAP analysis. Representative fluorescence images show the fluorescence of CD80-GFP before, immediately after, and 120 s after photobleaching. Yellow rectangles indicate the photobleached region of interest. Scale bars = 10 μm. **(B)** Representative fluorescence recovery curves for the examined HeLa cells are plotted as fluorescence intensity vs. time. **(C)** The fraction of mobile CD80 for each cell type. Error bars represent means ± SEM. (n = 9 to 12). **(D)** Z-stacked fluorescence imaging of CD80-GFP-expressing, gelated HeLa cells (4 wt%) shows filamentous patterns attributable to complexation between CD80 and actin cytoskeleton. Scale bar = 10 μm.

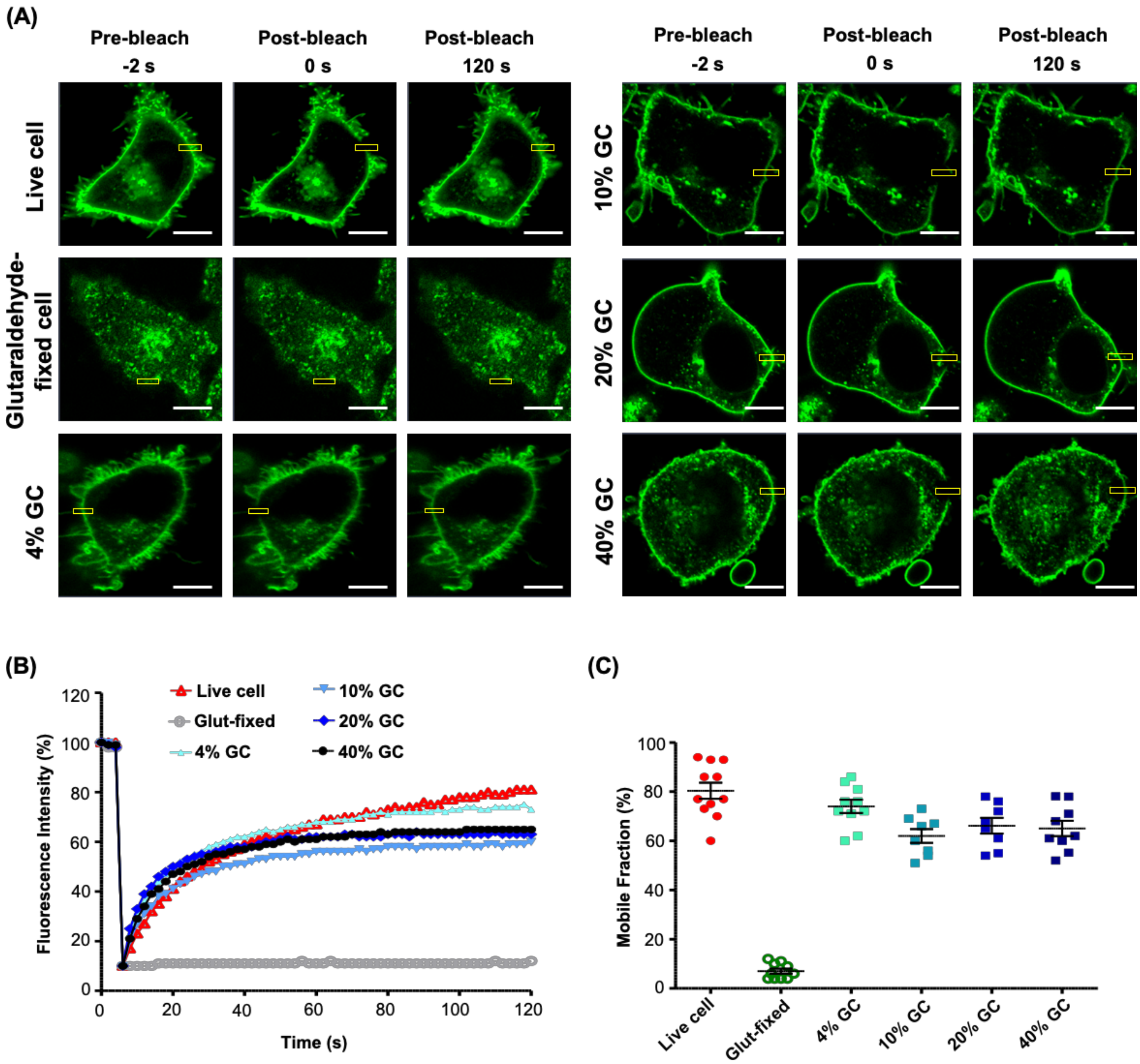

Supplementary Figure 11. Glycosylphosphatidylinositol (GPI)-anchored proteins are mobile on GCs. (A) HeLa cells transfected with EGFP-GPI plasmid were fixated with glutaraldehyde or gelated with different concentrations of PEG-DA prior to FRAP analysis. Representative fluorescence images show the fluorescence of EGFP-GPI before, immediately after, and 120 s after photobleaching. Yellow rectangles indicate the photobleached region of interest. Scale bars = 10 µm. **(B)** Representative fluorescence recovery curves for the examined HeLa cells are plotted as fluorescence intensity vs. time. **(C)** The fraction of mobile GPI for each cell type. Error bars represent means ± SEM. (n = 9 to 12).

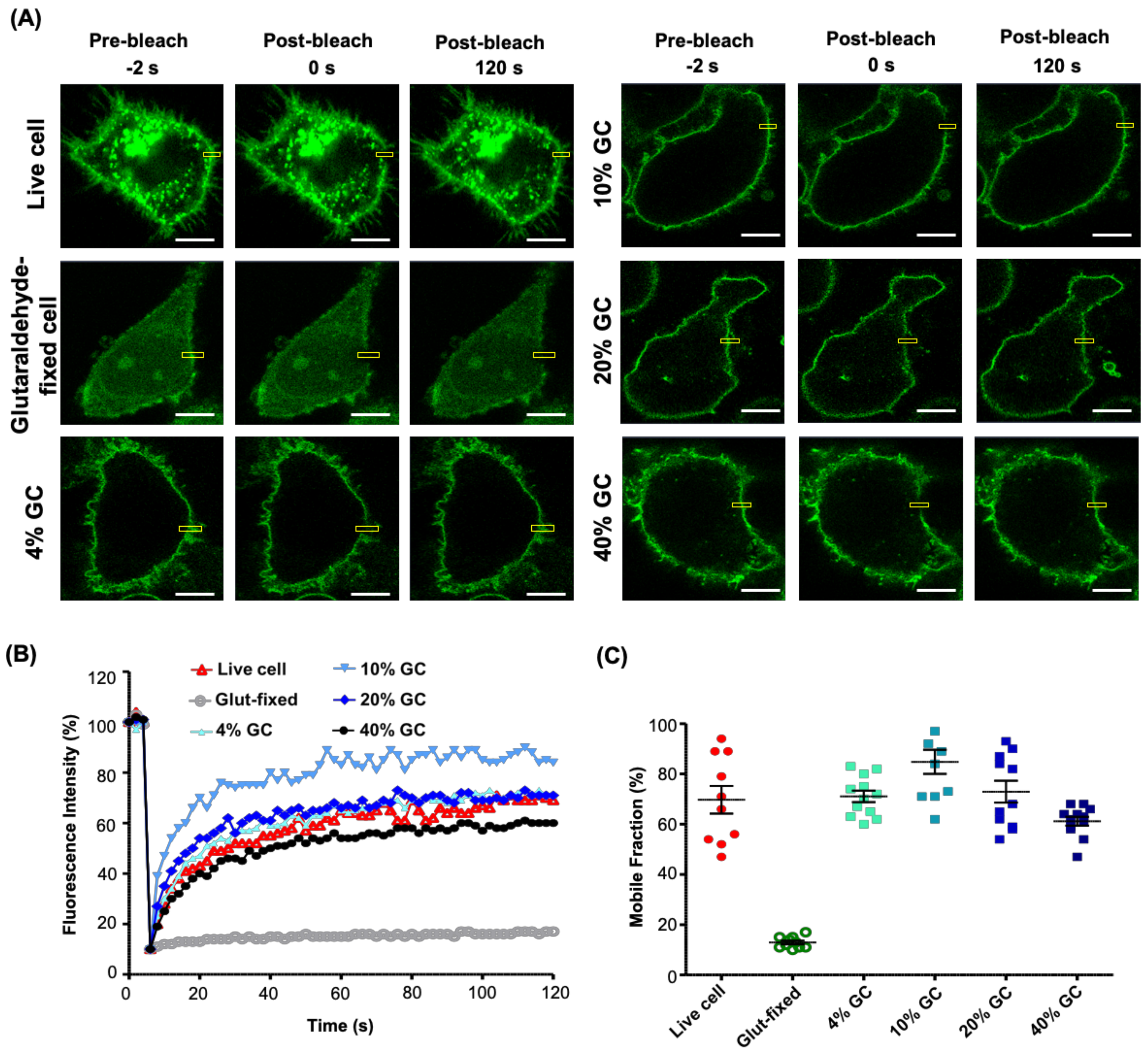

Supplementary Figure 12. Transferrin receptor (TfR) proteins are mobile on GCs. (A) HeLa cells transfected with TfR plasmid were fixated with glutaraldehyde or gelated with different concentrations of PEG-DA prior to FRAP analysis. Representative fluorescence images show the fluorescence of TfR before, immediately after, and 120 s after photobleaching. Yellow rectangles indicate the photobleached region of interest. Scale bars = 10 µm. **(B)** Representative fluorescence recovery curves for the examined HeLa cells are plotted as fluorescence intensity vs. time. **(C)** The fraction of mobile TfR for each cell type. Error bars represent means ± SEM. (n = 10 to 12).

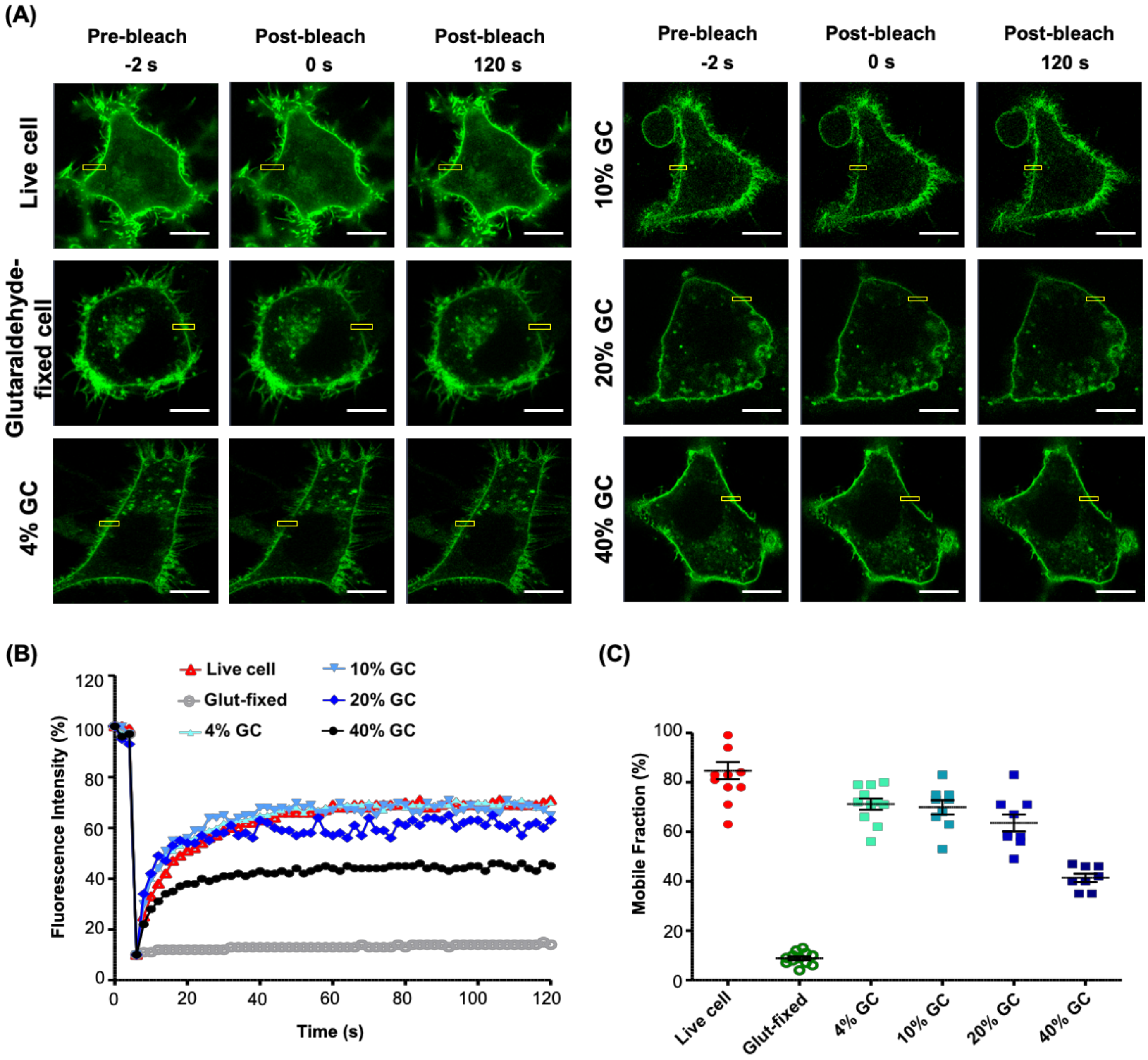

Supplementary Figure 13. Lyn kinase proteins are mobile on GCs. (A) HeLa cells transfected with Lyn kinase-GFP plasmid were fixated with glutaraldehyde or gelated with different concentrations of PEG-DA prior to FRAP analysis. Representative fluorescence images show the fluorescence of Lyn kinase before, immediately after, and 120 s after photobleaching. Yellow rectangles indicate the photobleached region of interest. Scale bars = 10 μm. **(B)** Representative fluorescence recovery curves for the examined HeLa cells are plotted as fluorescence intensity vs. time. **(C)** The fraction of mobile Lyn kinase for each cell type. Error bars represent means ± SEM. (n = 8 to 10).

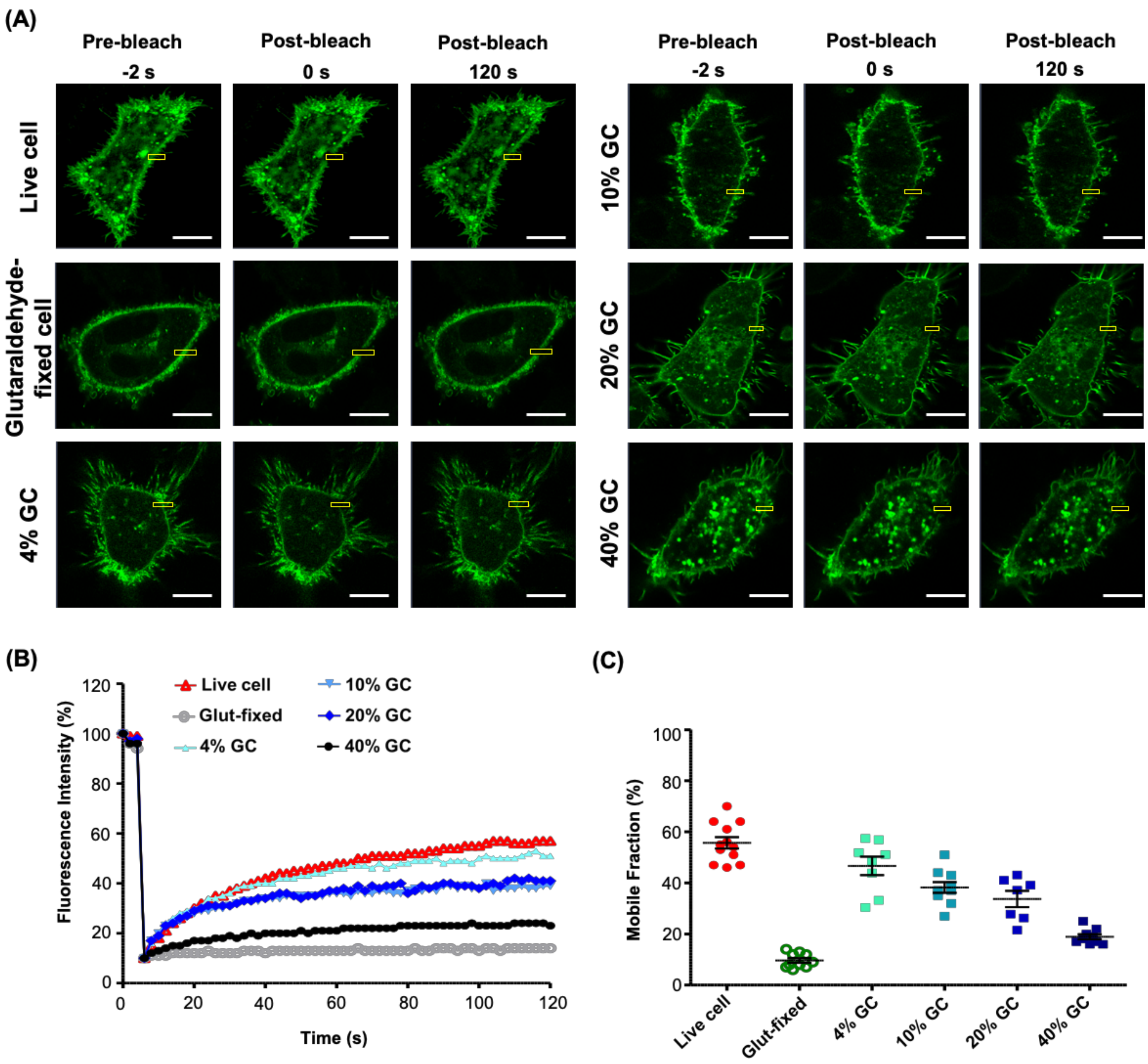

Supplementary Figure 14. EGFR proteins are mobile on GCs. (A) HeLa cells transfected with EGFR-GFP plasmid were fixated with glutaraldehyde or gelated with different concentrations of PEG-DA prior to FRAP analysis. Representative fluorescence images show the fluorescence of EGFR before, immediately after, and 120 s after photobleaching. Yellow rectangles indicate the photobleached region of interest. Scale bars = 10 µm. **(B)** Representative fluorescence recovery curves for the examined HeLa cells are plotted as fluorescence intensity vs. time. **(C)** The fraction of mobile EGFR for each cell type. Error bars represent means ± SEM. (n = 7 to 12).

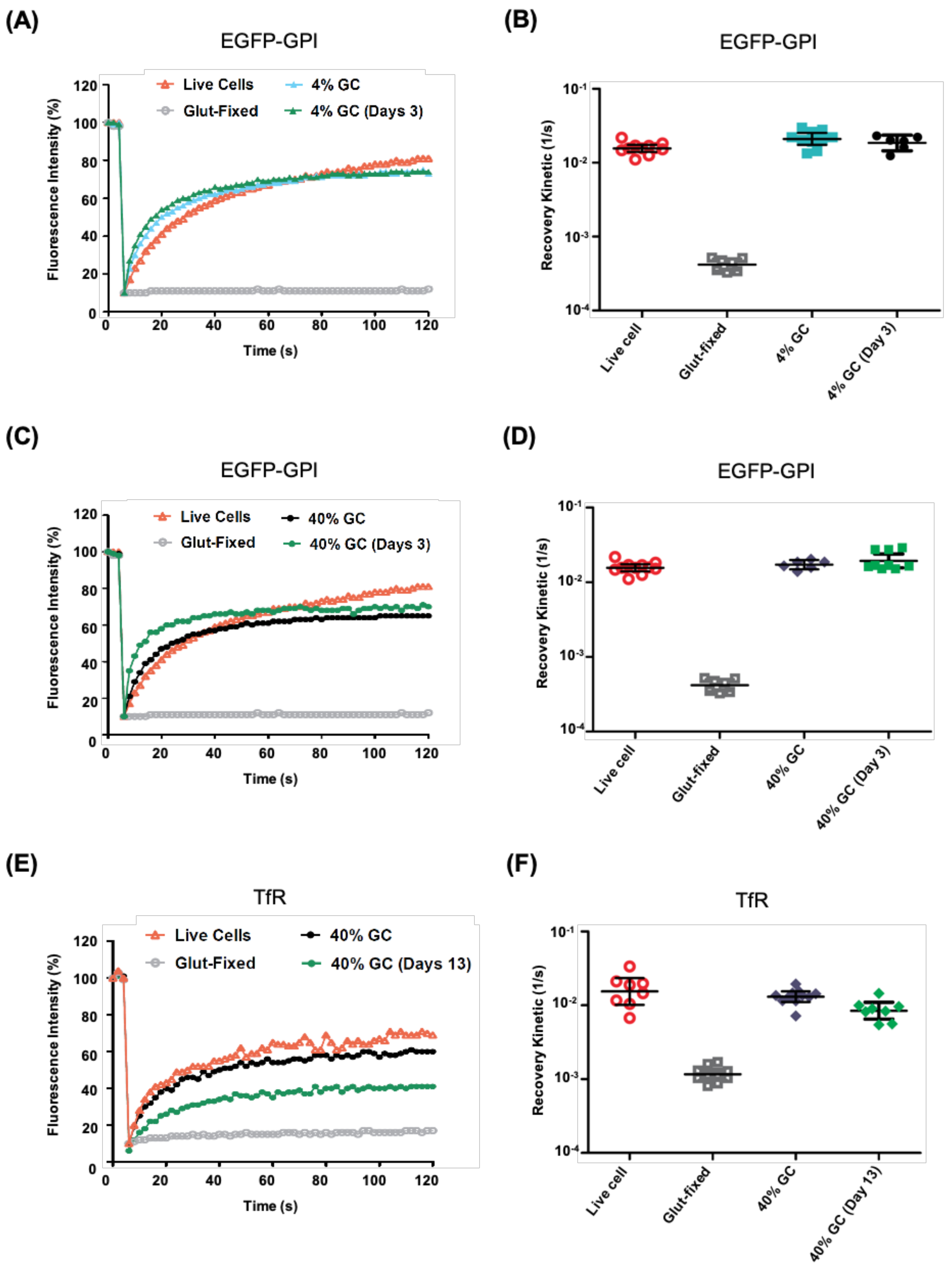

Supplementary Figure 15. Membrane protein mobility is preserved on GCs for extended period. HeLa cells transfected with EGFP-GPI **(A, B, C, and D)** and TfR **(E and F)** plasmids were gelated with 4 wt% or 40 wt% of PEG-DA and stored in PBS at 4C for the indicated time periods. **(A, C, and E)** Representative fluorescence recovery curves for the examined proteins are plotted as fluorescence intensity vs. time. **(B, D, and F)** The recovery kinetics of EGFP-GPI and TfR for the different cell types. Error bars represent means ± SEM. (n = 7 to 12).

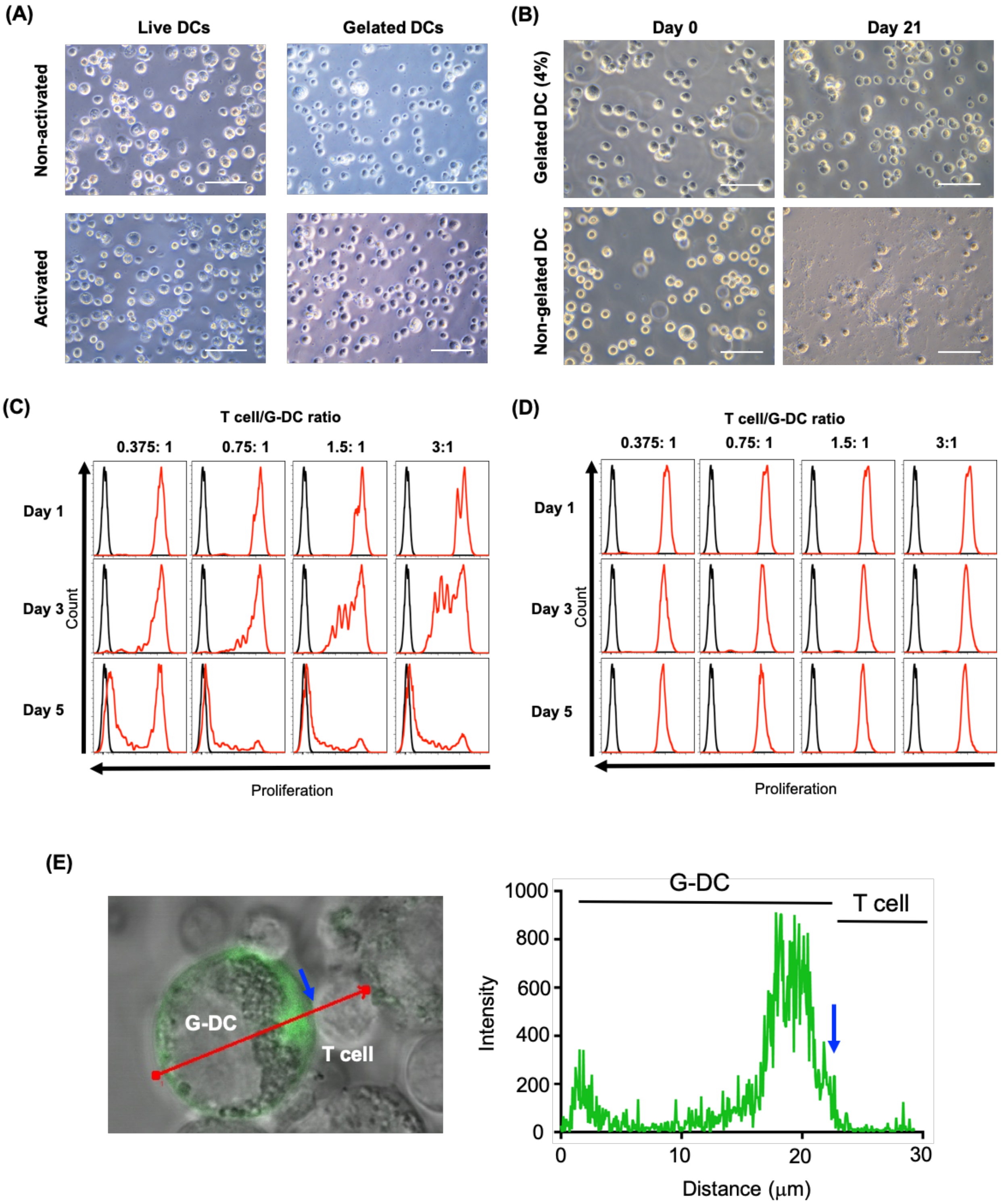

Supplementary Figure 16. Gelated dendritic cells (G-DCs) trigger the expansion of antigen-specific T cells. (A) Microscopic observation shows G-DCs (4 wt% PEG-DA) have similar morphology to live DCs. Scale bars = 50 μm. **(B)** The G-DCs showed little morphological changes whereas the non-gelated DCs disintegrated after 21 days. Scale bars = 50 μm. For the T-cell expansion study, both **(C)** activated and **(D)** non-activated G-DCs were co-cultured with antigen-specific CD8+ T cells stained with carboxyfluorescein diacetate succinimidyl ester (CFSE). Each culture condition contains a fixed number of G-DCs at 8×10^4 per well. T cell expansion was monitored on day 1, 3, and 5 at various T cell to G-DC ratios. Unstained T cells are plotted in black as a reference. **(E)** Fluorescence quantification of CD80 clustering at the G-DC/T cell interface. Red line indicates the cross-section for fluorescence intensity analysis. Blue arrow indicates the site of G-DC/T cell interaction.

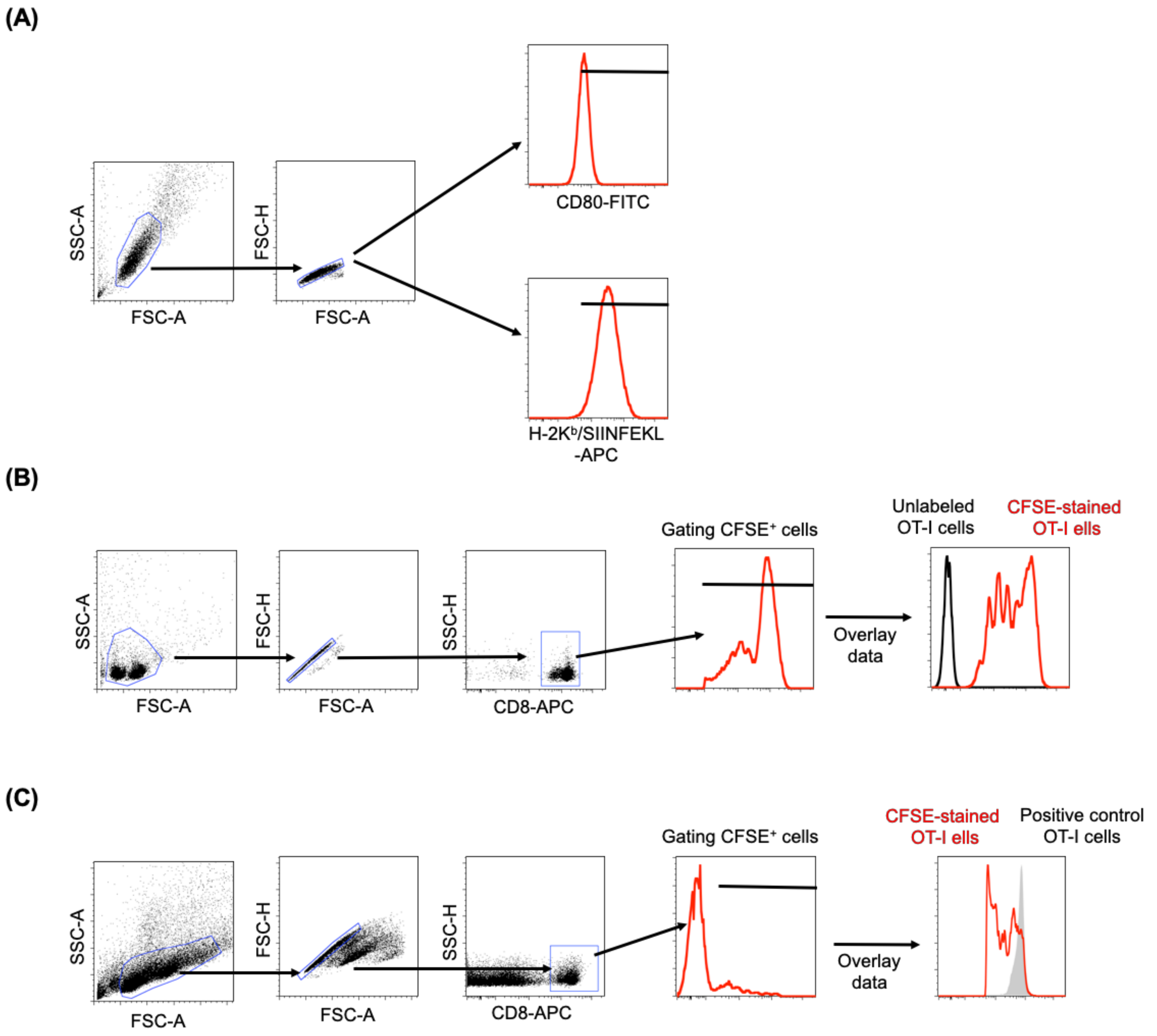

Supplementary Figure 17. Gating strategies for cytometric analyses. (**A**) Gating strategy to detect CD80 and H-2K^{b}/SIINFEKL expression for *the in vitro* assay presented on Figure 5B-E. (**B**) Gating strategy to measure the level of CFSE dilution in T cells for the *ex vivo* proliferation assay presented on Figure 5F and 7A, B. (**C**) Gating strategy to measure the level of CFSE dilution in T cells for the *in vivo* proliferation assay presented on Figure 7C-F.
