# Supplementary material for: Intracellular hydrogelation preserves fluid and functional cell membrane interfaces for biological interactions
Source: Nat Commun. 2019 Mar 5;10:1057. doi: 10.1038/s41467-019-09049-5 (PMC6401164; doi:10.1038/s41467-019-09049-5)
Supplement: Supplementary file 3 — Description of Additional Supplementary Files [file 41467_2019_9049_MOESM3_ESM.pdf]

Description of Additional Supplementary Items

File Name: Supplementary movie 1
Description: TIRF imaging of CD80-GFP on 4 wt% gelated cells.

File Name: Supplementary movie 2
Description: TIRF imaging of CD80-GFP on 10 wt% gelated cells.

File Name: Supplementary movie 3
Description: TIRF imaging of CD80-GFP on 20 wt% gelated cells.

File Name: Supplementary movie 4
Description: TIRF imaging of CD80-GFP on 40 wt% gelated cells.

File Name: Supplementary movie 5
Description: TIRF imaging of CD80-GFP on glutaraldehyde-fixed cells.

File Name: Supplementary movie 6
Description: TIRF imaging of CD80-GFP on non-gelated control cells.

File Name: Supplementary movie 7
Description: TIRF imaging of CD80-GFP on live cells.

File Name: Supplementary movie 8
Description: Fluorescence imaging of a gelated antigen-presenting cell interacting with CD8 T cells with the cognate T-cell receptor.
