# Supplementary material for: Intracellular hydrogelation preserves fluid and functional cell membrane interfaces for biological interactions
Source: Nat Commun. 2019 Mar 5;10:1057. doi: 10.1038/s41467-019-09049-5 (PMC6401164; doi:10.1038/s41467-019-09049-5)
Supplement: Supplementary file 12 — Reporting Summary [file 41467_2019_9049_MOESM12_ESM.pdf]

nature research

Corresponding author(s): Che-Ming Jack Hu

Last updated by author(s): Feb 2, 2019

Reporting Summary

Nature Research wishes to improve the reproducibility of the work that we publish. This form provides structure for consistency and transparency in reporting. For further information on Nature Research policies, see Authors & Referees and the Editorial Policy Checklist.

Statistics

For all statistical analyses, confirm that the following items are present in the figure legend, table legend, main text, or Methods section.

n/a	Confirmed	
☐	☒	The exact sample size (*n*) for each experimental group/condition, given as a discrete number and unit of measurement
☐	☒	A statement on whether measurements were taken from distinct samples or whether the same sample was measured repeatedly
☐	☒	The statistical test(s) used AND whether they are one- or two-sided<br>*Only common tests should be described solely by name; describe more complex techniques in the Methods section.*
☒	☐	A description of all covariates tested
☒	☐	A description of any assumptions or corrections, such as tests of normality and adjustment for multiple comparisons
☐	☒	A full description of the statistical parameters including central tendency (e.g. means) or other basic estimates (e.g. regression coefficient) AND variation (e.g. standard deviation) or associated estimates of uncertainty (e.g. confidence intervals)
☐	☒	For null hypothesis testing, the test statistic (e.g. *F*, *t*, *r*) with confidence intervals, effect sizes, degrees of freedom and *P* value noted<br>*Give P values as exact values whenever suitable.*
☒	☐	For Bayesian analysis, information on the choice of priors and Markov chain Monte Carlo settings
☒	☐	For hierarchical and complex designs, identification of the appropriate level for tests and full reporting of outcomes
☒	☐	Estimates of effect sizes (e.g. Cohen's *d*, Pearson's *r*), indicating how they were calculated

Our web collection on statistics for biologists contains articles on many of the points above.

Software and code

Policy information about availability of computer code

Data collection	Flow cytometry data was collected using FACSDiva software (v6.2). ELISA data was collected using SkanIt Software (v3.2). Eastic moduli were collected using JPK NanoWizard 3.
Data analysis	Statistical analyses were performed using GraphPad Prism software (v7). Flow cytometry data was analyzed using FlowJo software (v10). Image data was analyzed using ZEISS ZEN 2.3 (blue edition) and ImageJ (v1.52). ELISA data was collected using SkanIt Software (v3.2)

For manuscripts utilizing custom algorithms or software that are central to the research but not yet described in published literature, software must be made available to editors/reviewers. We strongly encourage code deposition in a community repository (e.g. GitHub). See the Nature Research guidelines for submitting code & software for further information.

Data

Policy information about availability of data

All manuscripts must include a data availability statement. This statement should provide the following information, where applicable:

- Accession codes, unique identifiers, or web links for publicly available datasets
- A list of figures that have associated raw data
- A description of any restrictions on data availability

All data generated or analysed during this study are included in this published article and its supplementary information files.

Field-specific reporting

Please select the one below that is the best fit for your research. If you are not sure, read the appropriate sections before making your selection.

☒ Life sciences ☐ Behavioural & social sciences ☐ Ecological, evolutionary & environmental sciences

Life sciences study design

All studies must disclose on these points even when the disclosure is negative.

Sample size	Sample sizes were estimated to achieve 90% power for detection of significant differences in tumor volume between groups based on means and standard deviations estimated in preliminary studies and were consistent with sample sizes used in similar reports in the literature.
Data exclusions	No data were excluded from the analyses.
Replication	Attempts to replicate all the data were successful.
Randomization	Mice were randomly sorted into treatment and control groups.
Blinding	In vivo T cell expansion studies were performed by independent researchers who were blinded as to treatment group assignment.

Reporting for specific materials, systems and methods

We require information from authors about some types of materials, experimental systems and methods used in many studies. Here, indicate whether each material, system or method listed is relevant to your study. If you are not sure if a list item applies to your research, read the appropriate section before selecting a response.

Materials & experimental systems

n/a	Involved in the study	
☐	☒	Antibodies
☐	☒	Eukaryotic cell lines
☒	☐	Palaeontology
☐	☒	Animals and other organisms
☒	☐	Human research participants
☒	☐	Clinical data

Methods

n/a	Involved in the study	
☒	☐	ChIP-seq
☐	☒	Flow cytometry
☒	☐	MRI-based neuroimaging

Antibodies

Antibodies used	Antibodies used in flow cytometry were obtained from Biolegend or eBioscience against: CD3(BioLegend, 145-2C11, #100306), CD80 (eBioscience, clone Ly-53, #11-0801-81), MHC class I-SIINFEKL (eBioscience, 25-D1.16, #17-5743-80), CD8a (eBioscience, 53-6.7, #100712). Flow cytometric antibodies were used at a 1:100 dilution.
Validation	Flow cytometric antibodies were validated by Biolegend or eBioscience. Related data is shown on the manufacturer website.

Eukaryotic cell lines

Policy information about cell lines

Cell line source(s)	Hela and JAWSII cells were obtained from American Type Culture Collection (ATCC; Manassas, VA, USA)
Authentication	All cell lines used were not authenticated by the authors
Mycoplasma contamination	All cell lines used in this paper tested negative for mycoplasma.
Commonly misidentified lines (See ICLAC register)	No commonly misidentified cell lines were used.

Animals and other organisms

Policy information about studies involving animals; ARRIVE guidelines recommended for reporting animal research

Laboratory animals	Experiments and handling of mice were conducted under federal, state, and local guidelines and with approval by the Academia Sinica Institutional Animal Care & Utilization Committee, Academia Sinica, Taipei, Taiwan. Six to eight week old B6 mice were purchased from BioLASCO Taiwan Co., Ltd.
Wild animals	The study did not involve the use of wild animals.
Field-collected samples	The study did not involve samples collected from the field.

Ethics oversight	All experimental protocols for animals required the approval and oversight by the Academia Sinica Institutional Animal Care & Utilization Committee.

Note that full information on the approval of the study protocol must also be provided in the manuscript.

Flow Cytometry

Plots

Confirm that:

☒ The axis labels state the marker and fluorochrome used (e.g. CD4-FITC).

☒ The axis scales are clearly visible. Include numbers along axes only for bottom left plot of group (a 'group' is an analysis of identical markers).

☒ All plots are contour plots with outliers or pseudocolor plots.

☒ A numerical value for number of cells or percentage (with statistics) is provided.

Methodology

Sample preparation	Spleen samples were harvested from sacrificed mice, placed in RPMI1640 complete medium with 10% fetal bovine serum (FBS) . Tissues were mechanically disrupted using a syringe and a 40 µm cell strainer. After samples were then passed through a 40 um cell strainer, cells were exposed to RBC lysing buffer (BD) for 5 minutes and then resuspended in RPMI1640 complete medium with 10% FBS. After washed with medium, samples were stained with CFSE (for 5 minutes) or the appropriate antibodies (for 30 minutes) in the dark, washed, and resuspended in medium for flow analysis. In order to harvest OT-I T cells, the Mouse CD8a+ T Cell Isolation Kit (BD Biosciences, #19853A) was used.
Instrument	FACSCanto, BD.
Software	Data collection: FACSDiva v6.2. BD and Data Analysis: FlowJo v10, Tree Star, Inc.
Cell population abundance	No sorting was performed.
Gating strategy	Representative gating strategies are shown in the Supplementary Figure 17. Cell debris were excluded from sample analysis by FSC-A/SSC-A discrimination. Doublets were excluded by FSC-A/FSC-H discrimination. For cell surface marker assay, gates were drawn to demarcate positive cell populations by the levels of fluorescence in negative controls (isotype antibodies). For proliferation assay, CFES positive cell populations were determined by unstained group.

☒ Tick this box to confirm that a figure exemplifying the gating strategy is provided in the Supplementary Information.
